# Microarray patch for HIV prevention and as a multipurpose prevention technology to prevent HIV and unplanned pregnancy: an assessment of potential acceptability, usability, and programmatic fit in Kenya

**DOI:** 10.3389/frph.2023.1125159

**Published:** 2023-04-24

**Authors:** Sammy Gakero Gachigua, Robinson Karuga, Anne Ngunjiri, Courtney Jarrahian, Patricia S. Coffey, Maggie Kilbourne-Brook, Lilian Otiso

**Affiliations:** ^1^ Research, LVCT Health, Nairobi, Kenya; ^2^ Medical Devices and Health Technologies, PATH, Seattle, WA, United States

**Keywords:** microarray patch, HIV PrEP, multipurpose prevention, contraception, Kenya, health product development, acceptability, microneedle patch

## Abstract

**Background:**

Microarray patches (MAPs), a novel drug delivery system, are being developed for HIV pre-exposure prophylaxis (PrEP) delivery and as a multipurpose prevention technology (MPT) to protect from both HIV and unintended pregnancy. Prevention technologies must meet the needs of target audiences, be acceptable, easy to use, and fit health system requirements.

**Methodology:**

We explored perceptions about MAP technology and assessed usability, hypothetical acceptability, and potential programmatic fit of MAP prototypes using focus group discussions (FGD), usability exercises, and key informant interviews (KII) among key populations in Kiambu County, Kenya. Adolescent girls and young women (AGYW), female sex workers (FSW), and men who have sex with men (MSM) assessed the usability and acceptability of a MAP prototype. Male partners of AGYW/FSW assessed MAP acceptability as partners of likely users. We analyzed data using NVivo, applying an inductive approach. Health service providers and policymakers assessed programmatic fit. Usability exercise participants applied a no-drug, no-microneedle MAP prototype and assessed MAP features.

**Results:**

We implemented 10 FGD (4 AGYW; 2 FSW; 2 MSM; 2 male partners); 47 mock use exercises (19 AGYW; 9 FSW; 8 MSM; 11 HSP); and 6 policymaker KII. Participants reported high interest in MAPs due to discreet and easy use, long-term protection, and potential for self-administration. MAP size and duration of protection were key characteristics influencing acceptability. Most AGYW preferred the MPT MAP over an HIV PrEP-only MAP. FSW saw value in both MAP indications and voiced need for MPTs that protect from other infections. Preferred duration of protection was 1–3 months. Some participants would accept a larger MAP if it provided longer protection. Participants suggested revisions to the feedback indicator to improve confidence. Policymakers described the MPT MAP as “killing two birds with one stone,” in addressing AGYW needs for both HIV protection and contraception. An MPT MAP is aligned with Kenya's policy of integrating health care programs.

**Conclusions:**

MAPs for HIV PrEP and as an MPT both were acceptable across participant groups. Some groups valued an MPT MAP over an HIV PrEP MAP. Prototype refinements will improve usability and confidence.

## 1. Introduction

In Kenya, the most recent estimated prevalence of HIV among adults was 4.9% in 2019 (1, 2), marking it as the country with the twelfth highest rate of HIV globally. In the same year, the estimated HIV prevalence for women aged 15–49 was more than twice as high as that for men aged 15–49 (6.6% vs. 3.1%). HIV infection rates among young people (15–24) accounted for 35% of new infections, with two-thirds of cases among adolescent girls and young women (AGYW) (3, 4). Also, the most recent national statistics (from 2016) showed an HIV prevalence of 18.2% among men who have sex with men (MSM) and 29.3% among female sex workers (FSW) (5). Reducing HIV infection rates among these populations is crucial for HIV epidemic control.

Likewise, unintended pregnancies in Kenya continue to be a public health burden. Although Kenya had a high contraceptive prevalence rate of about 58% for married women in 2020 (6), national survey data demonstrated that unmet need for family planning was highest among young women 20–29 years old (33%), followed by adolescent girls 15–19 years old (23%) (7). The high proportion of sexually active AGYW with unmet need for family planning in Kenya has led to a high number of unintended or mistimed pregnancies, unsafe abortions, and maternal deaths (8–10). Women, particularly AGYW, who face a persistent unmet need for contraception tend to have a higher risk of HIV infection (11–13). The high incidence of HIV among AGYW is exacerbated by low uptake of HIV prevention methods, such as pre-exposure prophylaxis (PrEP) (14, 15).

New drug delivery systems are being developed to address the challenges users experience (16–18) with PrEP delivery through daily oral pills. For example, a microarray patch (MAP) is being developed to deliver an antiretroviral (ARV) for HIV PrEP, as well as alongside a hormonal contraceptive as a multipurpose technology (MPT) for women. These ARV MAPs in development have the potential to offer protection for 1 month to 3 months, depending on the active pharmaceutical ingredient (API). The MAPs have the potential for easy, discreet, and self-administered protection that could improve uptake of and adherence to HIV PrEP. Qualitative research in 2016 with South African women and health care providers indicated that an ideal HIV PrEP solution should be discreet, long acting (3–6 months), highly effective, possible to self-administer, and protect users against not only HIV but also other sexually transmitted infections and pregnancy (19). A 2019/2020 assessment in South Africa and Uganda that explored user/stakeholder preferences regarding the MAP for HIV PrEP and as an MPT (20) generated recommendations for refinements to the MAP prototype, including the feedback indicator, to improve ease of use. Using the refined MAP prototype, we conducted this early-stage product development assessment in Kenya to continue exploring user and stakeholder needs and preferences for product features that could influence acceptability, usability, and programmatic fit for a MAP delivering HIV PrEP and as an MPT.

## 2. Materials and methods

**Figure 1:**
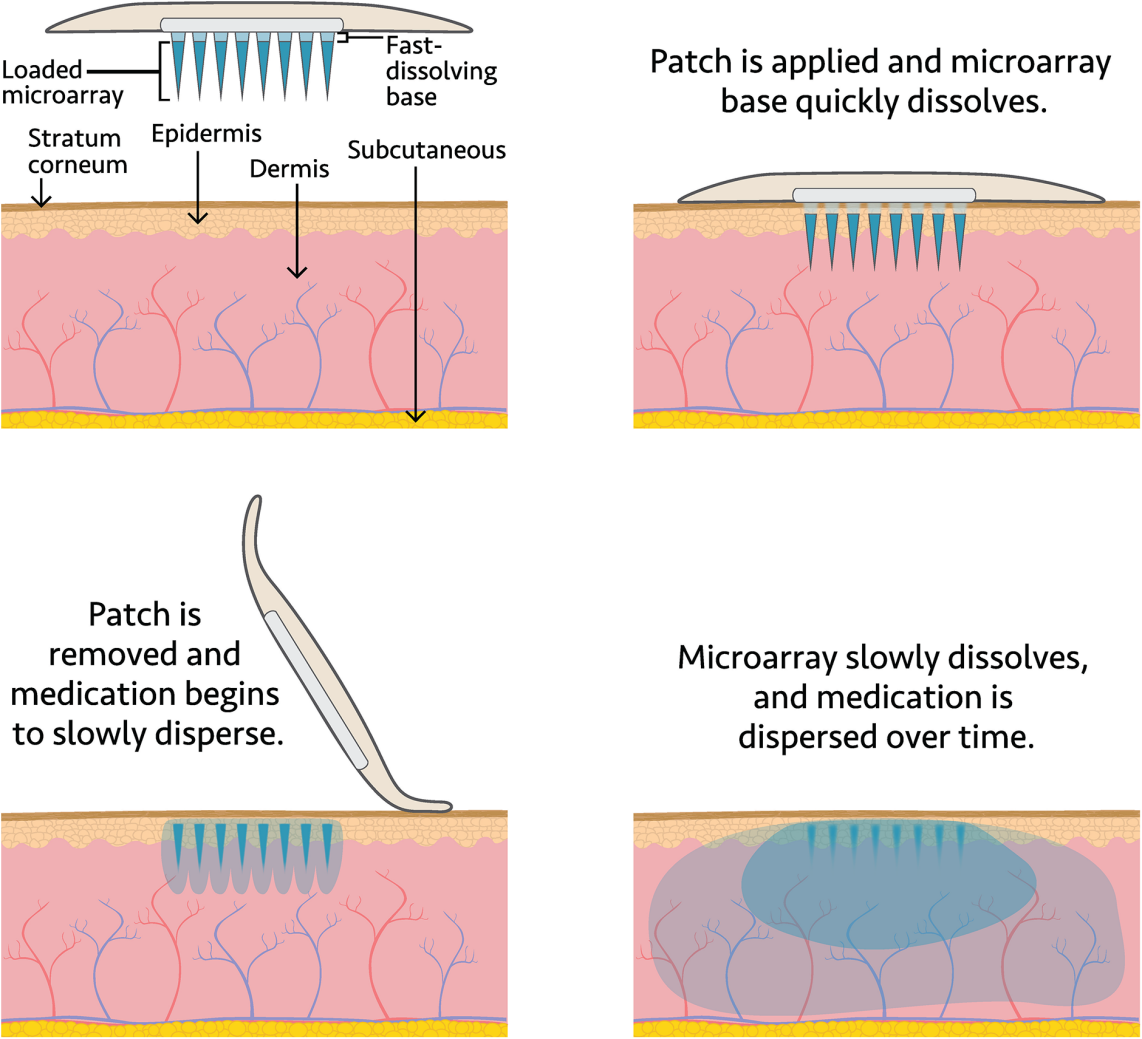
Dissolving MAP drug delivery system.

The ARV MAPs in development are designed to have multiple arrays, each containing hundreds of tiny (<1 mm) microneedles. Each array on the MAP has a corresponding dome above it that the user would depress to apply the patch to the skin; collectively, the domes serve as a “feedback indicator” to confirm successful application. The MAP projections would gently pierce the skin and begin to dissolve. First, the base of the projections dissolve, separating from the patch backing (Figure 1). After a specified wear time, the MAP backing and feedback domes would be removed and discarded. Next, the projections would fully dissolve in the skin, releasing the API into systemic circulation (21, 22). The targeted wear time for the HIV PrEP and MPT MAP is less than 20 min, at which point the MAP's adhesive layer would be discarded.

**Figure 2:**
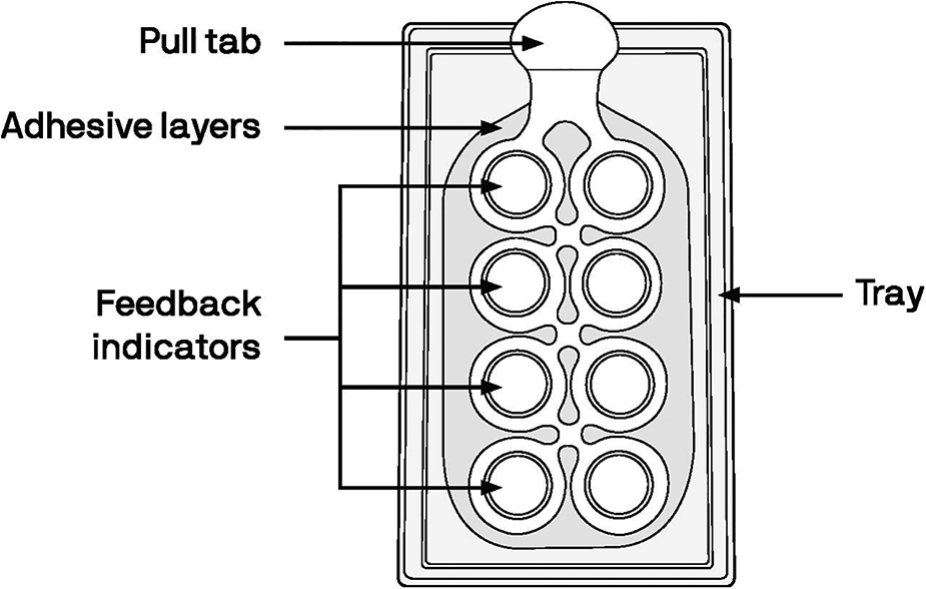
Diagram of the MAP prototype used in this assessment; dimensions were 14.5 cm × 6.25 cm (5.75 inches by 2.5 inches).

The MAP prototype used in this assessment (Figure 2) was representative of the aforementioned MAP currently being developed to deliver an ARV for HIV PrEP and as an MPT for delivery of both an ARV and a hormonal contraceptive. The MAP prototype had 8 feedback indicator domes indicating where microneedle arrays would be located (if it were a marketed product containing drugs). After the MAP prototype was applied to the skin, the user pressed on each dome until the domes inverted. The MAP prototypes used in this assessment were “looks like/feels like” prototypes—they did contain any microneedles and did not contain any drugs.

### 2.1. Study design

This descriptive exploratory study was conducted in Kenya, a country known as a leader in PrEP rollout, but where barriers continue to exist for currently available HIV prevention methods (23, 24). Within Kenya, Kiambu County was selected as the study site because it has a large proportion of AGYW, FSW, and MSM. Kiambu County hosts a high concentration of institutions of higher learning and corollary large populations of AGYW. The proximity of Kiambu County to Nairobi has resulted in a high rate of urbanization and related increase in the study populations of interest (25). In addition, the county provides access to peri-urban and rural settings. Importantly, service delivery points that serve AGYW, FSW, and MSM with HIV testing services were accessible and interested in study collaboration, as they are all owned and managed by LVCT Health.

The objectives of the study were to assess (a) usability of a MAP prototype (no drug or microneedles) for ease of use and design features, (b) hypothetical acceptability of MAP technology for HIV PrEP and/or as an MPT, and (c) potential programmatic fit of MAP technology within the Kenya health care system. Study participants included adolescent girls (AG, 15–17 years) and young women (YW, 18–24 years); FSW; MSM; male sexual partners of AGYW and FSW; health service providers; and national and county policymakers and managers. MSM assessed the MAP prototype for HIV prevention only.

Sampling for qualitative data collection considered the homogeneity of the populations and followed general guidance around the sample sizes required to reach saturation (26). The research team worked with staff at the five participating health facilities to select AGYW, FSW, male sexual partners of AGYW and FSW, and MSM participants for focus group discussions (FGD) *via* purposive typical case sampling whereby every third client from each of the target populations was asked to participate. Male sexual partners of YW were recruited *via* referral from their partners. We also purposively sampled national stakeholders and county-level health managers with experience in PrEP and contraceptive service provision and commodity management to assess the potential programmatic fit of MAPs for HIV PrEP and as an MPT.

We based our sample size (*n* = 14 per user group) for the mock use exercise on general guidance about appropriate sample sizes for usability testing (27). Because self-care products in Kenya, such as HIV self-testing (28), are usually delivered initially in health facilities where providers are able to assist, we also engaged providers to evaluate usability of the prototype *via* a simulated use exercise in which they were asked to provide additional support to mock clients.

### 2.2. Data collection and analysis

Primary data collection included a mock use exercise with a MAP prototype followed by in-depth interviews and a self-administered questionnaire with AG, YW, FSW, MSM, and health service providers. In addition, FGD were held with AGYW, FSW, male partners of AGYW and FSW, and MSM. Participants in these two data collection efforts were distinct from one another. Finally, key informant interviews were conducted with county and national stakeholders. In both data collection efforts, user perceptions and preferences about product features that could affect acceptability were explored, including MAP size, duration of protection, site of application, wear time, feedback indicator, and packaging. The mock use exercise consisted of orienting the participants to the MAP prototype through an informed consent process. Participants who consented were given instructions for use (IFU) (Supplementary material S1) and a MAP prototype and were asked to follow the instructions to apply the MAP on their body. During this activity, the participants were encouraged to “think out loud” and describe what they were doing. Simultaneously, a research assistant observed the mock use to document use errors, difficulties, close calls, and surprises across all steps of MAP application (handling, opening package, using instructions, practicing applying the patch, activating the feedback indicator, removing the patch). An in-depth interview was conducted to capture user perspectives after mock use, including a survey in which participants rated perceived importance and relative satisfaction with MAP features. Data collection methods are summarized in Table 1.

**Table 1:** Data collection methods, by study objective.

Study objective	User group	Data collection methods used
Usability of MAP prototypes	AG, YW, FSW, MSM, and health service providers	• Mock use exercise in which participants used the MAP prototype according to the instructions for use; researchers followed a standardized observation checklist to record correct use, use errors, close calls, difficulties, surprises; video recorded or photographed; duration of 15–30 min • In-depth interviews after mock use: audio recorded; duration of 45 min • Self-administered questionnaire in which participants ranked the MAP features and level of satisfaction with each feature in order of importance; duration of 15 min
Hypothetical acceptability of MAP technology for HIV PrEP and/or as an MPT	AGYW, FSW, male partners of AGYW and FSW, and MSM	Focus group discussions: audio recorded; duration of up to 90 min
Programmatic fit of MAP technology within the Kenya health care system	Health service providers and national/county-level stakeholders	Key informant interviews: audio recorded; duration of up to 45 min

AGYW, adolescent girls and young women; FSW, female sex workers; MAP, microarray patch; MSM, men who have sex with men; PrEP, pre-exposure prophylaxis.

The data collection instruments were translated into Kiswahili and pretested to identify ambiguity and clarify language. The FGD guide and semi-structured interview questionnaire were pretested with AGYW, FSW, MSM, and male partners at drop-in centers and DREAMS program sites in Nairobi County. The key informant interview questionnaire was pretested with providers and stakeholders in facilities in Nairobi County that did not participate in this study.

Data were collected in “safe spaces” identified by the facilities (where they usually meet confidentially with clients to discuss health issues). When a safe space was not available, clients were asked to suggest a meeting place within their community and interviewers assessed the location prior to the meetings to check whether it was conducive for data collection according to interview and research ethics requirements.

Qualitative data were transcribed verbatim in Microsoft Word (Microsoft Corporation, USA), translated as necessary, and analyzed applying an inductive approach using NVivo™ R (QSR International Pty Ltd; Doncaster, Australia). A coding framework was developed, initially based on the study objectives and then expanded in a data analysis workshop. Coding and qualitative data analysis were done collaboratively by the research team, noting comparisons (where appropriate) for the different target populations.

Quantitative data (ranking of MAP features and satisfaction from the self-administered questionnaire and mock use exercise observation checklist) were analyzed using Microsoft Excel version 2016 or a rainbow spreadsheet (29) with filters for user populations and usability steps. Data were cleaned and reviewed after entry into the spreadsheets; incomplete, inaccurate, or irrelevant data were identified and rectified after consultation with at least two members of the research team.

### 2.3. Ethics approval

The AMREF Ethics and Scientific Review Committee granted ethics approval for this study (approval number: P770/2020). Letters confirming approval to conduct the study were shared with county partners and clinic and drop-in center sites after meetings to sensitize the county representatives on the proposed study. The Kiambu County Health Research Unit also granted approval to conduct the research (reference number: KIAMBU/HRDU/22/03/08/RA_OTISO). Written informed consent was obtained for all study procedures, including audio, photo, and video recording, as warranted by each data collection method.

## 3. Results

Between February and April 2022, we collected data from 47 participants in a mock use exercise and conducted ten FGD and six stakeholder interviews (Table 2). The stakeholder interviews were conducted with three county managers and three national managers.

**Table 2:** Study participants, by user group and data collection method.

Data collection method	AGYW	FSW	MSM	Providers	Male partners of AGYW/FSW	Total number of participants
Mock use exercise	19	9	8	11	0	47
Focus group discussions	4	2	2	0	2	10 groups (total of 74 participants)
Stakeholder interviews						6

AGYW, adolescent girls and young women; FSW, female sex workers; MSM, men who have sex with men.

### 3.1. Usability of the MAP prototype

The research team observed 47 participants during simulated use of the MAP prototype (Table 3). Observations were recorded as either correct use or one of the four standardized categories employed in usability testing (30). The majority of the 47 participants reviewed the instructions before engaging in the mock exercise. The four participants who did not review the instructions experienced user errors. Successful completion of all steps in the mock use exercise was low for all user groups, ranging from 13% for MSM to 46% for providers. About a quarter of AG (29%), YW (25%), and FSW (22%) successfully accomplished the mock use exercise.

**Table 3:** Summary scores of mock exercise observations for all user groups (*n* = 47).

Observation	Successful use % (*n*)	User error % (*n*)	Difficulty % (*n*)	Close call % (*n*)	Surprise % (*n*)
Reviewed instructions	91 (43)	9 (4)	0	0	0
Fully understood instructions	30 (14)	70 (33)^a^	9 (4)_b_	0	0
Cleaned application site	47 (22)	53 (25)	0	0	0
Easily and correctly opened package	94 (44)	0	6 (3)	0	2 (1)^†^
Correctly peeled patch out of tray	98 (46)	0	0	2 (1)	0
Correctly placed MAP on skin	74 (35)	17 (8)	9 (4)	0	0
Understood feedback indicator	64 (30)	32 (15)	4 (2)	0	0
Crushed all domes	66 (31)	19 (9)	15 (7)	0	0
Comfortable wearing MAP	89 (42)	9 (4)	2 (1)^‡^	0	0
Understood wear time of 10 min	57 (27)	26 (12)	17 (8)	0	0
Successfully removed MAP	70 (33)	13 (6)	17 (8)	0	0
Understood MAP disposal instructions	79 (37)	21 (10)	0	0	0

MAP, microarray patch.

Use error: User action or lack of user action while using the MAP that leads to a different result than what is intended by the manufacturer or expected by the user.

Close call: User almost commits a use error while performing a task but recovers in time to avoid making the use error.

Use difficulty: Although users did not commit a use error, they might have difficulty performing the task (e.g., user hesitating, spending a long period of time on a task, requesting help or expressing difficulties).

Surprise: User action or lack of user action while using the medical device that was not expected by the researcher.

^a^ Experienced one or multiple user errors during the mock exercise.

^b^ Experienced user errors and demonstrated difficulty in accomplishing the mock exercise, with two participants showing extreme difficulty.

^†^ Participant used teeth to open package.

^‡^ Participant seemed nervous and later reported that she thought the MAP was an HIV test kit.

The most problematic task observed for all population groups except providers was cleaning of the application site (both user error and difficulty). For AGYW, the second highest number of user errors and difficulties was observed for understanding wear time. For FSW and MSM, the second highest number of user errors and difficulties was observed for pressing down firmly on all feedback indicator domes, one at a time, until each dome crushed. Health service providers were observed to have the most challenges with pressing down firmly on all indicator domes one at a time until each dome crushed (use errors and difficulties) and removing the MAP layer from the skin (difficulty only). Two FSW showed extreme difficulty when using the MAP; one mistook the MAP for an HIV test kit and the other was not literate.

**Figure 3:**
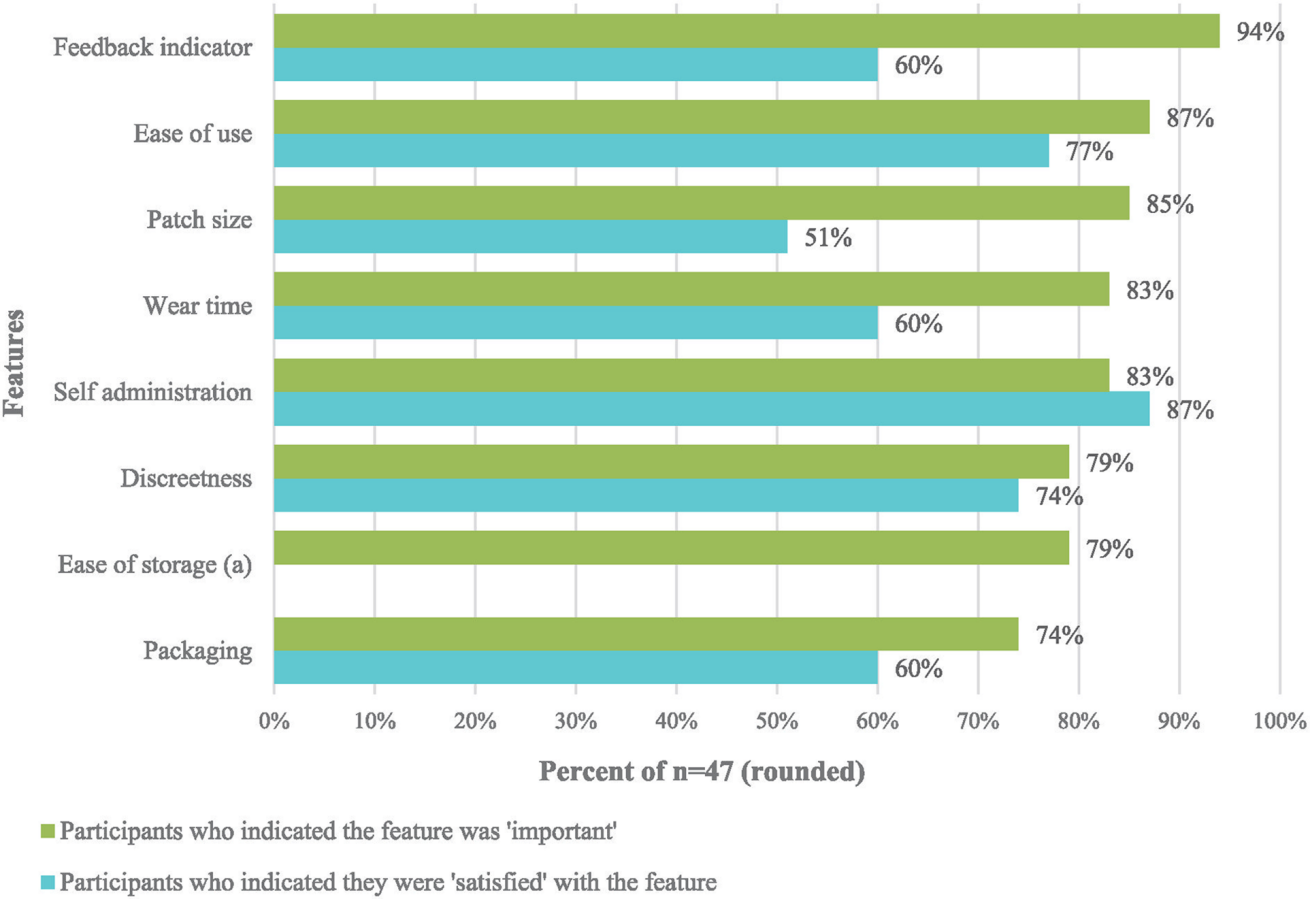
Differences between “important” and “satisfied” for MAP features after mock use by AGYW, FSW, MSM, and health service providers (*n* = 47).

After the mock use exercise, participants reported their perceived importance (“not important”, “undecided”, or “important”) and satisfaction (“dissatisfied”, “neutral”, or “satisfied”) about a set of MAP features (Figure 3 and Supplementary material S2). The feedback indicator, patch size, and wear time were the features with the largest gaps between importance and satisfaction, indicating product design alignments that will need to be made in future product iterations.

### 3.2. Hypothetical acceptability of a MAP for HIV PrEP or as an MPT

All FGD participants (AGYW, FSW, MSM, male partners) and mock use participants AGYW, FSW, MSM, providers) expressed their willingness to use a MAP for either HIV protection or as an MPT when it became available. Men shared opinions from the perspective of their own hypothetical use of an HIV PrEP MAP and perspectives about women using HIV PrEP MAP or MPT MAP. The convenience of the method and longer projected wear time were noted as advantages particularly when compared to existing methods of protection (Table 4). Conversely, a few male partners noted that despite their positive perception of the MAP, they could be suspicious that somebody using it might have an HIV infection.

**Table 4:** Illustrative acceptability of the MAP technology, by user group.

User group	Illustrative quote
Adolescent girls and young women	*“I would prefer patch because once you administer it the drugs get into the body. The drugs ok, you know us ladies if you go somewhere and you are late home you call mum and tell her that you may not be able to make it you will be hosted by somebody else, in that case you will miss your drugs because you have left it at home and with this it is in you.”* AG, mock use [IDIAG002]
Female sex workers	*“Because, it is cool; once you place it, that is it; it is not like the PrEP oral drug that you might sometimes forget to swallow; this is good*.*”* FSW, mock use [IDIFSW001] “*It is better to use this patch, because this patch is even easy to apply on the body anywhere. And then even when you use it, there is no one who is going to know whether there is anything that you are using.”* FSW, mock use [IDIFSW001]
Male partners	*“I think this one is better because not everyone normally can bear the burden of taking pills but this one is better since you just apply it on your skin.”* Male sexual partner, FGD [FGDMSP001]
Men who have sex with men	*“I like it because it is not like PrEP that you have to take every day, you can take it for a week, two weeks, monthly…yeah.”* MSM, mock use [IDIMSM001]
Providers	*“The patch is much better than the oral PrEP. I have interacted with adolescent girls and young women. From my experience condoms are not consistently used. Still the patch will be better.”* Provider, mock use [IDIHSP001]

Preferences around the MAP design feature set varied among user groups (Table 5). More detailed results about each feature are also discussed below.

**Table 5:** Summary of MAP feature preferences, by user group.

User group	Size	Wear time, in minutes	Duration of protection, in months	Two most preferred sites of application
Adolescent girls and young women	Acceptable	1–10	1	Thigh, arm
Female sex workers	Smaller	5–10	3–12	Upper arm, lower arm, thigh
Male partners	Smaller	10	<1 (7 days)	Arm, thigh
Men who have sex with men	Smaller	5–10	At least a week	Upper arm, lower arm, chest
Providers	Smaller	2–15	1–3	Inside upper arm

#### 3.2.1. MAP size

In general, AGYW reported that the current size of the MAP was acceptable. For example:

“*I haven’t seen anything that is wrong with the size because it can easily be covered*.” YW, mock use exercise [IDIYW002].

Service providers and FGD participants felt the size of the MAP should be smaller. They suggested the MAP should be small enough to be carried easily, possibly in a pocket or purse.

“*It can be smaller, so that when you put it in your handbag, someone should not see that it is something funny. It should look like something smart, small.”* FSW, FGD [FGDFSW001]

“*It's too long…some clients would not like to be seen by their partners…it would raise some alarm or queries.”* Provider, mock use exercise [IDIHSP001].

“*I wish it was smaller. It is big in that when you carry it you can’t put it in a smaller purse.”* Male partner of AGYW, FGD [FGDMSP001].

In particular, FGD participants recommended the MAP be about 7 cm × 7 cm (about 2.75 inches square), similar in size to a nicotine patch, deworming medicine patch, or Elastoplast (bandage).

#### 3.2.2. Wear time

Most participants preferred a short period of wear time (≤10 min) because it would be reasonable, convenient for busy lifestyles, and allow discreet application. Adolescent girls preferred the shortest wear time. A few participants mentioned that they preferred longer wear times (30–60 min or even 24 h), primarily to ensure that the drug had been fully delivered.

“*One minute. It should be something that works fast.”* AG, FGD [IDIAG001].

“*10 min is good for me. It is a reasonable time, anyone can get those ten minutes. Let's say you go to work in the morning and wake up at 7am, you can still be able to get the ten minutes to apply the patch. Ten minutes is okay.*” YW, mock use [IDIYW003].

“*I think those 10 min are okay, because even if you are at home, and you have bathed and put it on, by the time you finish getting ready, those 10 min will have passed. I think that time is okay those 10 min will not prevent you from doing your normal business.”* FSW, FGD [FGDFSW001].

“*If am applying it to a client it should take ten minutes. It should not take long since there might be other clients waiting.”* Provider, mock use [IDIHSP002].

“*Because you can set apart five to ten minutes knowing that you are applying some medication and then after ten minutes you go and do your chores.”* MSM, mock use [IDIMSM001].

#### 3.2.3. Duration of protection

The majority of AGYW preferred at least a 1-month duration of protection because it offered greater convenience and flexibility within their sexual and reproductive life. All providers proposed that the duration of protection should last between 1 and 3 months. One provider explained that shorter periods would not cure the problem of non-compliance with PrEP, and concomitantly, longer periods would reduce the burden of regular client visits to health facilities.

“*I would say so to reduce clients from coming back to the facility. My cry is actually for the clients since as a provider I will always be here. As you can see calling clients is sometimes hectic. They say they are not available on certain days.”* Provider, mock use [IDIHSP002].

FSW stated their preference for longer periods of protection than AGYW. It appeared that longer periods were favored because the FSW had had experience with injectable contraceptives (3-month protection) and/or contraceptive implants that had much longer periods of protection.

“*In fact, not even for 1 month; if I find for a whole year, I can be very happy.”* FSW, FGD [FGDFSW001].

Providers and policymakers expressed preference for a longer-term duration of protection (3–6 months) to improve protection and simplify resupply.

However, MSM and FSW also said having even 1 week of protection would be better than oral daily pills for HIV protection.

#### 3.2.4. MAP application site

After mock use, most participants preferred the MAP application site to be on the thigh (large surface, discreet) or the forearm or upper arm (ease of access, convenient). In FGD, the three most preferred sites were the upper arm, lower arm, or thigh. AG also mentioned the stomach as a third preference and YW mentioned under the breast and the rib cage as being appropriate application sites. Most providers preferred the inside upper arm, similar to placement for contraceptive implants (ease of access, does not invade client's privacy). Some mock use participants (AGYW) were confused about where to apply the MAP and wanted more guidance.

### 3.3. Programmatic fit of the MAP within the Kenya health care system

There was a general consensus among stakeholders that the MAP technology, whether HIV only or as an MPT, was a revolutionary innovation whose introduction would be very timely within the Kenya health care system. Overall, stakeholders preferred the MPT MAP over the HIV-only MAP. Stakeholders noted that the MPT MAP aligned well with the integrated health services policy currently in place in Kenya (31).

“*I think the combined would work better because it will make the integration of service easier. That this is a PrEP and a family planning and then it is dealing with two birds with one stone. I like the integration part, because we are looking at integrating services and integrating HIV to other services. So, you are killing two birds with one stone. At least that it does so we don’t have to deal with issues of unwanted pregnancies and abortions and complications of unwanted pregnancies and everything so that's a plus for the women. As we know, we are dealing with two pandemics here, the pandemic of unintended pregnancies and the pandemic of HIV especially women between 15 and 24 years. So having a product that helps you to address those two things together, it is very beneficial. From our data we can see we are getting many new HIV infections in that age. Then we are currently focusing on service integration, integrating PrEP into SRH and SRH into PrEP. We are currently trying to put systems in place to actualize integration.”* Female, national-level key informant interview [IDIKII002].

Additionally, stakeholders noted that an MPT MAP would save client time, reduce stigma associated with HIV prevention by combining with less stigmatized family planning services, reduce the pill burden associated with taking oral PrEP and of taking multiple drugs at different times and the service provider/facility burden of dispensing them, and be attractive to AGYW who are keen to prevent pregnancy at the expense of HIV protection. The stakeholders felt that an MPT MAP would be received with high enthusiasm/interest among AGYW, FSW, and providers. On the other hand, it was noted that the HIV PrEP MAP would be preferred by male users and female clients who had intolerance for hormonal contraceptives.

In general, stakeholders perceived the MAP technology favorably and identified product benefits as being its potential for discreetness, ease of application, long-term protection, and self-administration. For example, the simplified administration offered by the MAP would provide flexibility in terms of who can deliver the MAP and where it can be distributed/delivered, thus helping improve the overall efficiency of health services. Stakeholders mentioned that the MAP technology could likely improve PrEP uptake by redressing the major problems associated with oral PrEP.

“*Uptake of PrEP in the country is still low. Let us be honest, oral PrEP uptake is still low and one key challenge is the small things the end users raised were not addressed. The issue of the rattling sound of the tablet, the tablet is so big, the color looks like an ARV, you know, those small things. So, I’m very excited about this new product.”* Female, national-level key informant interview [IDIKII002].

Stakeholders agreed that the minimum duration of protection should be 3 months, to coincide with the recommended HIV retesting period and the schedule for injectable contraception. They also felt that MAPs should come in different durations of protection (3 months, 6 months, and 1 year) to address the needs of different users and that the package color should indicate duration.

Stakeholders identified multiple access points for a MAP product, including both facility-based and home-based self-care options.

“*For me, I see this product having a broad spectrum of delivery points. I see it beyond the current delivery point like for the oral PrEP. I see it as a self-empowering product which should be delivered at the comfort of somebody's home or even privacy. Just like a HIV self-test kit, buy in the chemist, go with it at home, when I am free, test.”* Female, national-level key informant interview [IDIKII002].

Several potential risks and/or unanswered questions were raised by stakeholders. These included the need for clarifying information about which drug(s) would be used in the MAP and the impact/safety; how to reverse the long-term MAP HIV prevention and contraceptive drugs in the body if they started having adverse effects on users; how the MAP feedback indicator would ensure that potential users have the optimal dosage of the prescribed drug(s); the sensation of using microneedles, including pain and side effects on skin; how to build user confidence that medication is actually delivered if no sensation; and the potential impact of repeated MAP applications (possible scarring). Stakeholders also raised the possibility of improper MAP use, both from the perspective of the technology and the intended user group (AG).

“*So, there is a risk of misuse because maybe of a client not understanding that if the duration of the product is 30 days. You see there is nothing being left there (nothing left inside the skin like the depo); how do you convince me that just that one application has left enough drugs to protect me for 30 days, you might find some people repeating the administration and that is a risk.”* Female, national-level key informant interview [IDIKII001].

“*I foresee this spilling over even in schools because it is very easy to administer. Those in school may opt for such a product and, as I had said, the policy currently for the in-school is age-appropriate information and abstinence, but because it is easy to administer this product, it will be easily administered even in school. We will not have a way of monitoring…how will we monitor that? So, I do not know how we will restrict this product to ensure that it is only maybe those children who can consent…in our country it is 18 years; it is only accessible to those above 18 years.”* Female, national-level key informant interview [IDIKII003].

A final concern, raised by policymakers, was the possible environmental impact of improper waste disposal.

“*Yes, plastic is harmful for the environment and that is why am suggesting there should be instructions on how to dispose it. We can also explain to the client how to dispose; is it burning it, or?”* Provider, mock use [IDIHSP002].

## 4. Discussion

In our study, all user groups expressed willingness to use MAPs as a stand-alone HIV prevention technology. Users showed a strong preference for MAP technology over other currently available HIV prevention methods (oral PrEP, condoms). Women, providers, and stakeholders expressed strong interest in an MPT MAP—sometimes in preference to a stand-alone HIV PrEP MAP. Some potential users also expressed preference for the contraceptive MAP concept over existing contraceptives because it would be easy to use, discreet, and self-administered. The strong interest in the MAP technology was related primarily to its potential for discreet use and self-administration, ease of application, and long-term protection. MAP size and duration of protection were seen as key characteristics influencing acceptability. Some participants—especially MSM/FSW, who preferred long-term protection—would accept a larger MAP if it provided longer protection. Most AGYW preferred the MPT MAP over an HIV PrEP-only MAP and preferred 1-month protection. This finding in support of an MPT option is consistent with the results of the Tablets, Ring, Injections as Options (TRIO) study in which young women aged 18–30 in Kenya and South Africa perceived high value for an MPT (32, 33), and preferred a longer duration as well as discreet protection (34, 35). Similarly, results from a discrete choice experiment among women and adolescents in South Africa reported likely limited uptake and health impact among adolescent women unless the new PrEP products also provide pregnancy protection (33, 36). An assessment of the potential for MPTs in Nigeria, South Africa, and Uganda also found that 93% of women surveyed preferred an MPT product to either an HIV-only or contraceptive-only product (37). Because Kenyan AGYW seeking contraception frequently have high HIV risk (38), an MPT option could be particularly beneficial for this user group. Policymakers in this study also noted that the MPT MAP could address needs of AGYW who are keen on avoiding pregnancy but also are at risk of HIV.

FSW often have overlapping burdens: high risk of HIV, unmet need for contraception, and increased likelihood of contracting a sexually transmitted infection (39). FSW saw value in both MAP indications and voiced a need for MPTs that protect from other infections besides HIV. FSW wanted duration of protection consistent with injectable contraception. Other user groups, such as women living with HIV, have also identified a longer-acting injectable as preferred over daily oral tablets when a multipurpose technology concept offered an antiretroviral for HIV treatment co-administered with a hormonal contraceptive (40). However, this may reflect respondent bias in that injectable contraception is well accepted in Kenya, thereby making it a familiar benchmark technology.

Male partner support can be an important influence on AGYW and FSW who are interested in using HIV PrEP (41–44). In this study, male partners were supportive of MAP use, although they mentioned that partner use of an MPT MAP might give rise to suspicions around partner fidelity and serostatus. Male partners of young Kenyan and South African women in the TRIO study, while being supportive of MPT use generally, also expressed similar concerns about product use disclosure (45).

MSM viewed the HIV PrEP MAP as being a viable option, which is noteworthy given that MSM in Kenya show low adherence to a daily PrEP regimen (46, 47). A recent programmatic surveillance of PrEP program rollout in Kenya also showed substantial missed opportunities for PrEP initiation for MSM, as well as high levels of PrEP discontinuation at 1 month (48). In other studies (49, 50), MSM have noted their preferences for longer-acting PrEP options. Use of novel delivery platforms such as the MAP could be important for MSM, because PrEP discontinuation is not uncommon.

Providers and stakeholders preferred an MPT MAP and expressed that an MPT MAP could ease workload in health facilities and is aligned with Kenya's policy of integrating health care programs. The MPT MAP was identified by stakeholders as a revolutionary technology that has the potential to “kill two birds with one stone.” Enthusiasm for MPTs has been documented by health care providers in Kenya and South Africa where one South African nurse explained that provision of an MPT to YW could “kill two birds with one stone” (51). This finding strongly parallels our study findings, with one stakeholder using the exact same language to illustrate their point. Regardless of type of MAP (i.e., HIV PrEP, MPT as well as a contraceptive MAP), stakeholders felt that the technology could ease the burden of existing methods for users, providers, and the health care system.

In this study, Kenyan participants evaluated a second-generation prototype design that had been refined based on participant experiences with earlier prototypes in South Africa and Uganda (20). The MAP prototype used in the Kenya assessment was designed to be scaled for different numbers of arrays, depending on API potency. The second-generation prototype had an improved feedback indicator that was optimized for ease of use through (a) reduced number of handling tabs; (b) larger arrays, enabling fewer domes to press; (c) material chosen for optimized inversion force; (d) refined dome design to ensure no rebound; and (e) cutouts to increase flexibility (to accommodate different body locations).

The mock use exercise identified several areas in need of further product iteration. Most participants (70%) across all user groups had some difficulty understanding the IFU, in part due to low literacy and poor comprehension of images. Participants recommended simplifying the language and making the graphics more distinct to improve clarity. Importantly, users need a MAP orientation and demonstration before use. This is similar to the MAP assessment in Uganda, where participants who were oriented to the MAP solely by the IFU experienced more difficulty using the MAP during mock use. In contrast, mock use participants in South Africa were recruited from FGD where they saw a demonstration of the MAP and had an opportunity to familiarize themselves with the device, resulting in a more successful user experience. Even with the refinements made to the current MAP prototype and IFU to improve clarity and ease of use, the Kenya results indicate that the potential user will benefit from an orientation and product demonstration before their first use.

Although most participants (74%) were able to place the MAP on the skin correctly, potential users across all groups wanted more guidance on exact placement of the MAP on the skin. YW displayed the most difficulty in this regard. Mitigation for this would be to integrate more direct counsel on freedom of choice for the application site when orienting the potential user to the device. Alternatively, a specific site could be identified as the best possible location and potential users could be instructed to apply in that location.

Across all groups, some participants (34%) struggled with activating the MAP feedback indicator by, for example, not crushing all domes or not pressing firmly enough to completely crush the domes. Providers were able to complete this step successfully, yet they felt that it took too much effort to crush the domes. This design issue could be remedied by providing more explanation on how the MAP works during orientation and/or in the IFU. Investigating the use of more pliable dome material and how it might result in reliable insertion of the MAP projections would be another potential option to address this concern.

Most users (73%) had difficulty understanding wear time of the device because they found interpreting the clock on the IFU challenging. Providers were able to interpret the clock correctly; however, they noted that it may be difficult for their clients. Using digital time in the IFU may help overcome this issue.

Some participants (30%) had difficulty removing the MAP layer, which could be resolved by providing a pull tab. Most participants (79%) understood instructions for device disposal, and some felt that disposal could be a health hazard. Information on safe disposal will be added to the IFU.

This study indicates that additional refinements are needed to optimize the MAP prototype, including the feedback indicator. These refinements could improve confidence in appropriate delivery, especially for low-literate and AGYW user groups, particularly if they have not had specific counseling about the MAP before use. Similar to results from the South Africa and Uganda assessments (20), these results from Kenya show a strong interest in a MAP as a drug delivery platform and desire for an MPT MAP that is long acting.

The use of mixed methods in this study strengthens the robust nature of the findings. The study is limited by the relatively small geographic distribution of participants (only from Kiambu County); however, participants from varied settings (urban, peri-urban, and rural) were included. Preferences about a contraceptive MAP were inferred from responses about an MPT MAP. Specific questions about a contraceptive MAP were deleted from data collection tools because results from the instrument pretesting indicated that no additional insights would be collected with contraceptive-specific questions. Data were analyzed with a focus on user group rather than these varied settings, so some additional nuance and learning may still be uncovered. Because the usability testing was not with a microarray patch containing microneedles, some skin reactions (from wearing the patch), side effects (e.g., pain) at the wear site could not be assessed thus biasing potential user acceptability more favorably. Additionally, many of the MSM and some of the FSW appeared to have been intoxicated when they participated in the mock use exercise, potentially affecting their ability to perform adequately. On the other hand, their level of sobriety may be representative of their real-life situations and data collected would reflect that reality. Nonetheless, engaging users and stakeholders in early-stage product development gives an opportunity to refine MAP design to better meet user needs. Users/stakeholders want to be part of the process of developing new products and moving them forward, and this type of mixed methods assessment offers an ideal opportunity for this involvement.

## 5. Conclusions

Participants reported high potential acceptability of MAP as a drug delivery system for both for HIV PrEP and as an MPT. Health service providers and policymakers felt MAP could be integrated into the HIV and family planning health care systems. Some potential target audiences seemed to value a MPT MAP over an HIV PrEP-only MAP—specifically, AGYW, health service providers, and policymakers. Reducing the overall MAP size and number of arrays would likely improve acceptability, the feasibility of which is dependent on successfully formulating a higher-potency ARV to be delivered by MAP. Further prototype modifications, such as refining the feedback indicator to provide greater confidence of successful application and instructing users when to remove the MAP, are recommended to improve confidence and acceptability.

## Data Availability

The original contributions presented in the study are included in the article/**Supplementary Material**, further inquiries can be directed to the corresponding author.
