# Supplementary material for: Microarray patch for HIV prevention and as a multipurpose prevention technology to prevent HIV and unplanned pregnancy: an assessment of potential acceptability, usability, and programmatic fit in Kenya
Source: Front Reprod Health. 2023 Apr 24;5:1125159. doi: 10.3389/frph.2023.1125159 (PMC10164997; doi:10.3389/frph.2023.1125159)
Supplement: Supplementary file 1 [file Table1.docx]

**Supplementary material 2**

As an elaboration on *Figure 3*, this table shows participant perceptions of the importance of and satisfaction with a set of MAP features (n=47; rounded percentages).

|  | ***[Satisfaction]*** | ***[Importance]*** | ***[Satisfaction]*** | ***[Importance]*** | ***[Satisfaction]*** | ***[Importance]*** |
| --- | --- | --- | --- | --- | --- | --- |
| **Feature** | **‘Satisfied’ % (n)** | **‘Important’  % (n)** | **‘Neutral’  % (n)** | **‘Undecided’  % (n)** | **‘Not satisfied’ % (n)** | **‘Not important’  % (n)** |
| Feedback indicator | 60% (28) | 94% (44) | 9% (4) | 6% (3) | 26% (12 ) | 0 |
| Ease of use | 77% (36) | 87% (41) | 11% (5) | 2% (1) | 6% (3) | 11% (5) |
| Patch size | 51% (24) | 85% (40) | 21% (10) | 0% (0) | 6% (3) | 15% (7) |
| Wear time | 60% (28) | 83% (39) | 15% (7) | 6% (3) | 19% (9) | 11% (5) |
| Self-administration | 87% (41) | 83% (39) | 4% (2) | 9% (4) | 4% (2) | 9% (4) |
| Discreetness | 74% (35) | 79% (37) | 9% (4) | 11% (5) | 11% (5) | 11% (5) |
| Ease of storage^i^ | - | 79% (37) | - | 4% (2) | - | 17% (8) |
| Packaging | 60% (28) | 74% (35) | 6% (3) | 6% (3) | 30% (14 ) | 19% (9) |
| *^I^ Satisfaction with Ease of storage was not recorded because the MAP was not stored.* | | | | | | |
