# Supplementary material for: Microarray patch for HIV prevention and as a multipurpose prevention technology to prevent HIV and unplanned pregnancy: an assessment of potential acceptability, usability, and programmatic fit in Kenya
Source: Front Reprod Health. 2023 Apr 24;5:1125159. doi: 10.3389/frph.2023.1125159 (PMC10164997; doi:10.3389/frph.2023.1125159)
Supplement: Supplementary file 2 [file Datasheet1.pdf]

Micro Array Patch Instructions for Use

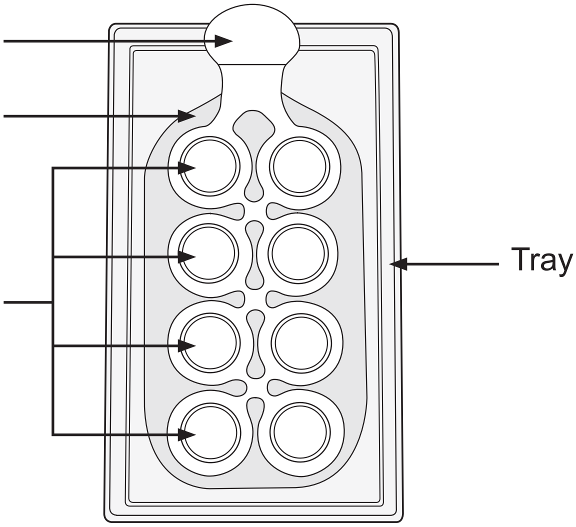

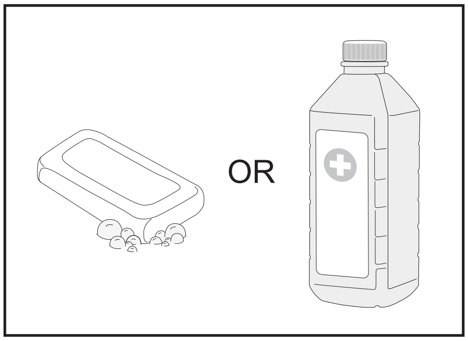

1. Clean skin where you will apply the patch (wash/dry or use alcohol wipe, if available).

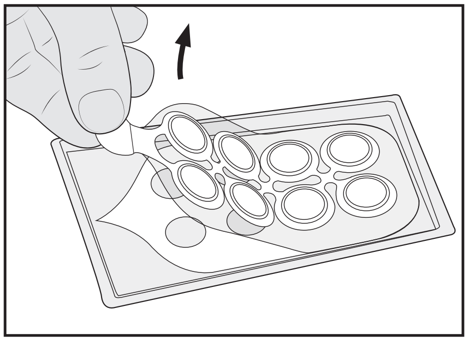

2. Remove feedback indicator and the patch layers together from tray using the pull tab.

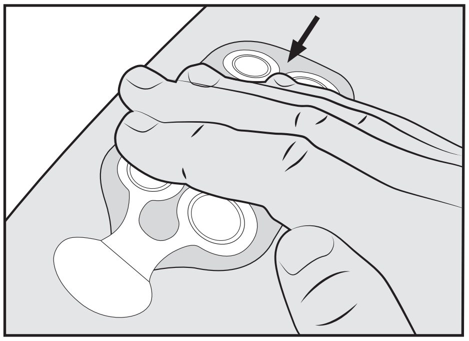

3. Apply to skin firmly, pressing adhesive onto skin.

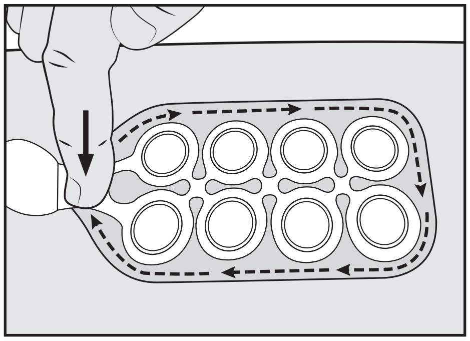

4. Press firmly around edges to ensure MAP layer sticks to the skin.

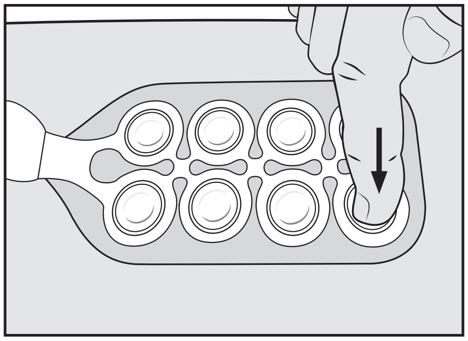

5. Press down firmly on all indicator dots one at a time until dome crushes.

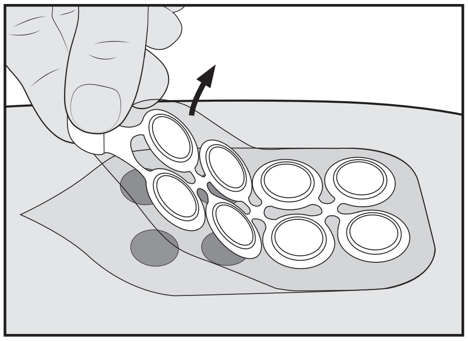

6. Pull tab to remove indicator layer, leaving MAP layer on the skin.

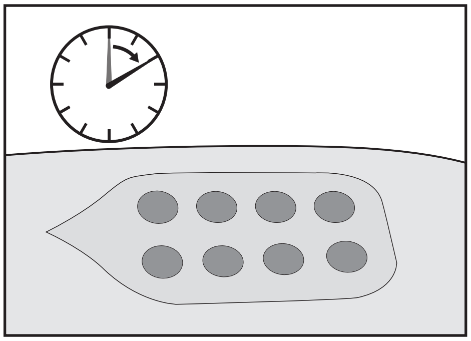

7. Leave MAP layer on skin for indicated time period.

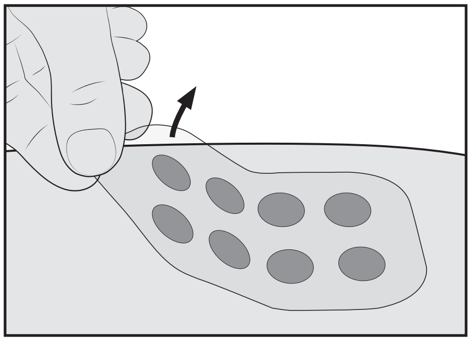

8. Remove MAP layer.

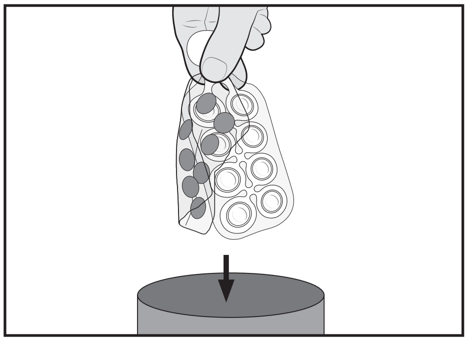

9. Dispose of MAPs and indicators.
